# Accuracy, Precision, Ease-Of-Use, and Cost of Methods to Test Ebola-Relevant Chlorine Solutions

**DOI:** 10.1371/journal.pone.0152442

**Published:** 2016-05-31

**Authors:** Emma Wells, Marlene K. Wolfe, Anna Murray, Daniele Lantagne

**Affiliations:** Department of Civil and Environmental Engineering, Tufts University, Medford, Massachusetts, United States of America; NIH, UNITED STATES

## Abstract

To prevent transmission in Ebola Virus Disease (EVD) outbreaks, it is recommended to disinfect living things (hands and people) with 0.05% chlorine solution and non-living things (surfaces, personal protective equipment, dead bodies) with 0.5% chlorine solution. In the current West African EVD outbreak, these solutions (manufactured from calcium hypochlorite (HTH), sodium dichloroisocyanurate (NaDCC), and sodium hypochlorite (NaOCl)) have been widely used in both Ebola Treatment Unit and community settings. To ensure solution quality, testing is necessary, however test method appropriateness for these Ebola-relevant concentrations has not previously been evaluated. We identified fourteen commercially-available methods to test Ebola-relevant chlorine solution concentrations, including two titration methods, four DPD dilution methods, and six test strips. We assessed these methods by: 1) determining accuracy and precision by measuring in quintuplicate five different 0.05% and 0.5% chlorine solutions manufactured from NaDCC, HTH, and NaOCl; 2) conducting volunteer testing to assess ease-of-use; and, 3) determining costs. Accuracy was greatest in titration methods (reference-12.4% error compared to reference method), then DPD dilution methods (2.4–19% error), then test strips (5.2–48% error); precision followed this same trend. Two methods had an accuracy of <10% error across all five chlorine solutions with good precision: Hach digital titration for 0.05% and 0.5% solutions (recommended for contexts with trained personnel and financial resources), and Serim test strips for 0.05% solutions (recommended for contexts where rapid, inexpensive, and low-training burden testing is needed). Measurement error from test methods not including pH adjustment varied significantly across the five chlorine solutions, which had pH values 5–11. Volunteers found test strip easiest and titration hardest; costs per 100 tests were $14–37 for test strips and $33–609 for titration. Given the ease-of-use and cost benefits of test strips, we recommend further development of test strips robust to pH variation and appropriate for Ebola-relevant chlorine solution concentrations.

## Introduction

Ebola is an enveloped, single-stranded RNA virus in the *Filoviradae* family that causes severe Ebola Virus Disease (EVD) in humans [1]. The case fatality rate ranges from 25–100% [2]. The disease begins abruptly and presents with a high fever, headache, muscle pain, weakness, diarrhea, and vomiting, along with a characteristic rash and hemorrhaging in some patients [3]. Prior to the 2014 West African outbreak, the largest EVD outbreak included a comparatively few (425) cases in Uganda in 2000–2001 [4]. The 2014 West African EVD outbreak was the first widespread outbreak; since December 2013 there have been 28,607 cases of EVD and 11,314 deaths, mostly within West Africa but spreading to ten countries including the United States and several European nations [5].

Ebola is transmitted by direct contact with infected humans or animals, or through indirect contact with objects or surfaces contaminated with bodily fluids of patients [6,7]. Disinfection is necessary to prevent transmission, and when chlorine is used Doctors without Borders (MSF), the Centers for Disease Control and Prevention (CDC), and the World Health Organization (WHO) all recommend the use of 0.05% (500 mg/L) solutions to disinfect living things (hands and people) and 0.5% (5,000 mg/L) solutions to disinfect non-living things (surfaces, personal protective equipment, dead bodies) [8–10]. These recommendations were initially created for use in Ebola Treatment Units (ETUs). However, the unprecedented magnitude of the current outbreak has led to evolving response strategies, and these recommendations have been widely applied in community settings [11,12].

Chlorine solutions are usually generated on-site, and therefore it is important for responders to test concentrations for quality control. There are three chlorine source compounds commonly used in EVD response to manufacture chlorine solutions: powdered calcium hypochlorite (HTH), granular sodium dichloroisocyanurate (NaDCC), and liquid sodium hypochlorite (NaOCl) [13,14]. For each of these compounds, disinfection efficacy can vary based on the concentration of the chlorine solution, contact time, temperature, pH, and presence of organic material [15,16]. Lower pH solutions are generally more efficacious than higher pH solutions [17,18], although the impact of pH in disinfection practices specific to EVD outbreaks has not been evaluated. The expected pH values of chlorine solutions used in EVD contexts vary from approximately 6–7 for NaDCC solutions, 10–11 for HTH and for stabilized NaOCl, 9 for generated NaOCl, and 7 for neutralized NaOCl [13]. We were unable to locate previous research investigating the performance of chlorine concentration test methods using different chlorine source compounds.

How to accurately and inexpensively test chlorine solution concentration was a concern for: 1) water, sanitation, and response staff in ETU settings [19] and 2) the United Nations Development Programme (UNDP), as they found chlorine solutions were increasingly manufactured in non-controlled settings, and stored for long periods of time [11]. User-friendly colorimetric test methods and strips commonly used to measure drinking water free chlorine residual in emergency response are able to measure concentrations of 0–4 mg/L, and have been found to vary in terms of accuracy, precision, ease-of-use, and cost [20]. However, these test methods are not appropriate for testing the higher Ebola-relevant chlorine solution concentrations of 500 and 5,000 mg/L unless modified.

In this study, we first identified chlorine concentration testing methods recommended for, or used in, EVD response. We then evaluated fourteen test methods for accuracy, precision, ease-of-use, and cost, using five chlorine solutions manufactured from three source chlorine compounds at two concentrations. The aim was to develop chlorine concentration testing recommendations for EVD outbreak response.

## Methods

To establish recommendations for chlorine concentration test methods appropriate for EVD outbreak use, we: 1) identified commonly-used source chlorine compounds and test methods by contacting organizations involved in EVD response and searching on-line materials; 2) prepared five different chlorine solutions at 0.5% and 0.05% and tested their concentrations with identified methods for accuracy and precision; 3) asked volunteers to use each method to assess usability; and 4) calculated testing cost. All research activities took place at the Environmental Sustainability Laboratory and classrooms at Tufts University in Medford, MA, United States. The study protocol was approved by the Social, Behavioral, and Educational Research Institutional Review Board at Tufts University.

### Test Method Selection

To select test methods that are appropriate for field conditions, 108 people from 37 different organizations were contacted for information on the source chlorine compounds and test methods used in EVD response. Emails were sent initially to the Ebola WASH Cluster mailing list, the Emergency Environmental Health mailing list, and personal emergency response contacts of the authors. The email was subsequently widely forwarded, including to the larger WASH Cluster mailing list. Additional online published literature [21] and the Ebola KnowledgePoint discussion forum [13] were also searched to identify test methods recommended or used.

### Accuracy and Precision

#### Chlorine Preparation

Five types of chlorine were prepared, all at both 0.05% and 0.5% concentrations: HTH, NaDCC, laboratory-grade pH-stabilized NaOCl (stabilized NaOCl), non pH-adjusted NaOCl produced with an electrochlorinator (generated NaOCl), and NaOCl produced with an electrochlorinator that was pH-neutralized via the addition of acetic acid (neutralized NaOCl). All solutions were prepared on the morning of testing using chlorine-demand free water from a Milli-Q Reference system (Milli-Q water) (EMD Millipore, Darmstadt, Germany). After preparation, chlorine concentrations were confirmed using Hach iodometric titration method 8209 (Digital Titration) (Hach Company, Loveland, CO), and adjusted if necessary until within 10% of the target concentration. Subsequently, solution pH was measured using a calibrated HI 9811–5 pH probe (Hanna Instruments, Woonsocket, Rhode Island).

HTH solutions were prepared by mixing commercially-available powder containing 65% available chlorine (Acros Organics, Pittsburgh, PA) with water. The solution was then allowed to settle for 24 hours in a covered, opaque HDPE bucket, and decanted. NaDCC solutions were prepared by dissolving Klorsept (formerly Aquatabs®) granules containing 50% available chlorine (Medentech Ltd, Wexford, Ireland) in water. Stabilized NaOCl was made by diluting a 5% laboratory-grade solution (RICCA Chemical, Arlington, TX) in water. Generated NaOCl solutions were produced by running a brine mixture containing non-iodized table salt (Stop & Shop Brand, Quincy, MA) and water through an AquaChlor on-site electrochlorinator for four hours (International Equipment & Systems, Inc., Miami, FL). Neutralized NaOCl solutions were produced by adding 25% laboratory grade acetic acid (Cole-Parmer, Vernon Hills, IL) to electrochlorinator produced solutions. In this process, pH was continuously measured using a HI 9811–5 pH probe and adjusted until a pH of 7 was reached.

#### Chlorine Testing

Each of the fourteen test methods was used according to the manufacturer’s instructions to measure chlorine concentrations of test solutions in quintuplicate. The methods used were grouped by type: 1) titration, 2) dilution followed by N,N diethyl-p-phenylene diamine (DPD) addition, and 3) test strips (Table 1). Titration methods test total chlorine, while DPD methods and test strips test free chlorine residual. In laboratory circumstances, as source chlorine compounds were diluted in demand-free water, total and free chlorine residual were equivalent.

**Table 1:** Test Methods Evaluated, with Range and Measurement Increment.

Test	Manufacturer	Test Method	Range (mg/L)	Measurement Increment (mg/L)
Digital Titration	Hach Company	Titration	20–70000	2.5
WataTest	Antenna	Titration	1000–7000	500
Pooltester (Cylinder Dilution)	Palintest	DPD	0–6	Low level: 0.1, High level: 0.5
Pooltester (Pipette Dilution)	Palintest	DPD	0–6	Low level 0.1, High level 0.5
Color Wheel (Cylinder Dilution)	Hach Company	DPD	0–3.4	0.2
Color Wheel (Pipette Dilution)	Hach Company	DPD	0–3.4	0.2
Indigo	Indigo Labs	Test Strip	0–10,000	0, 1000, 2500, 5000, 7500, 10000
Activate	Activate	Test Strip	0–10,000	0, 1000, 2500, 5000, 7500, 10000
Precision Extra	Precision Labs	Test Strip	0–10,000	0, 1000, 2500, 5000, 7500, 10000
Serim	Serim Monitor	Test Strip	100–750	100, 200, 350, 500, 750
Precision High	Precision Labs	Test Strip	0–1,000	0, 50, 100, 250, 500, 1000
WW Ultra I	Waterworks	Test Strip	0–750	0, 25, 100, 200, 300, 400, 500, 750
WW Ultra II	Waterworks	Test Strip	0–2,000	0, 25, 50, 200, 500, 800, 1100, 1500, 2000
InstaTest	LaMotte	Test Strip	0–800	0, 50, 100, 250, 500, 800

We evaluated two titration methods, Hach iodometric titration method 8209 (Digital Titration) (Hach Company, Loveland, CO) and WataTest (WataTest) (Antenna Technologies, Geneva, Switzerland) (Table 1). Both call for the addition of reagent(s) until a color change is visually observed. Digital Titration was used as a reference method because it is widely accepted as the most accurate method of chlorine testing available in the field, and was used for testing at both 0.05% of 0.5% concentrations (test range 1–70,000 mg/L). The performance of each kit was therefore compared to the digital titration method. Testing was performed by: adding solution with pipette and diluting to 50 mL with Milli-Q water in an Erlenmeyer flask; adding one powder sachet to acidify the sample; adding a potassium iodide solution to the acidified chlorine solution so that the reaction with chlorine liberates iodine in proportion to the chlorine in the solution; and titrating with a sodium thiosulfate cartridge using a starch indicator solution until the color changes from blue to clear. The titrator reading was multiplied by the appropriate digit multiplier to obtain results in mg/L. For 0.05% solution, 2 mL of solution was added and the 0.113 N sodium thiosulfate cartridge was used; for 0.5% solution, 4 mL of solution was added and the 2.0 N sodium thiosulfate cartridge was used. The WataTest method was used for the 0.5% solutions (test range 1000–6000 mg/L) by: using the plastic pipette provided to draw up 2 mL of solution; placing solution in a glass 20 mL beaker set on two white sheets of paper; filling the provided syringe with the WataTest reagent; slowly releasing one drop of the WataTest reagent from the syringe into the beaker, gently swirling the solution between each addition of a drop, and counting the number of drops released from the syringe until the solution color changed to clear. The number of drops was divided by two to obtain results in g/L and multiplied by 1,000 to obtain results in mg/L.

We evaluated two DPD free chlorine residual (FCR) test methods: Palintest Pooltester 610 (Pooltester) (Palintest, Gateshead, England), intended for use measuring chlorine in recreational pools, and Hach Chlorine Color Disk, model CN-66 (Color Wheel) (Hach Company, Loveland, CO), intended for use measuring chlorine in drinking water (Table 1). These methods are based on addition of DPD to solutions and comparison of resulting pink color to a standard chart. The test range for the Pooltester is 0–5 mg/L and for the Color Wheel is 0–3.4 mg/L. To use these kits, both 0.05% and 0.5% solutions were diluted with pipette dilution and graduated cylinder dilution. Pipette dilution (pipette or pip.) was used to dilute both 0.05% and 0.5% chlorine solutions to a concentration of 2 mg/L. A two-step dilution using graduated cylinders (cylinder or cyl.) was used to dilute solutions to 5 mg/L for the Pooltester and 2.5 mg/L for the Color Wheel. After each dilution, the Pooltester was used by: filling the chlorine portion of the test kit with solution, adding the provided DPD-1 free chlorine residual tablet, covering the kit, shaking it for 30 seconds, comparing the resulting solution color to the test kit color scale, and multiplying the result by the original dilution factor. The Color Wheel was used by: filling both provided tubes with 5 mL of chlorine solution, adding the DPD-1 free chlorine residual packet to one tube and swirling, placing both tubes in the test kit, holding the test kit up to the light for color comparison, and multiplying the resulting color match by the original dilution factor.

We evaluated eight test strip methods: five appropriate for 0.05% solutions, and three appropriate for 0.5% solutions (Table 1). Test strips are dipped in chlorine solution, and then the resulting color is matched to a standard to determine the free chlorine residual amount. For 0.5% chlorine solutions, we tested: 1) Precision Labs extra high-level chlorine test strip (Precision Extra) (Precision Labs, Cottonwood, AZ); 2) Indigo Lab 10K chlorine test strip (Indigo) (Indigo Labs, Waterloo, ON); and, 3) Activate high-level chlorine test strip (Activate) (Merlin, OR).

Precision Extra, Indigo, and Activate all required the same steps: dipping strip in the solution for 1 second, removing the strip, waiting for 30 seconds, and then comparing within 10 seconds to the color chart. All test strips had the same color chart range, from brown to yellow, with color indicators for the following chlorine concentrations: 0, 1,000, 2,500, 5,000, 7,500, and 10,000 mg/L.

For 0.05% chlorine solutions, we tested: 1) Serim Monitor for chlorine test strip (Serim) (Elkhart, IN); 2) Lamotte InstaTest free chlorine high-range test strip (InstaTest) (Chestertown, MD); 3) Waterworks Free Chlorine Check Ultra High (WW Ultra I) test strip (Rock Hill, SC); 4) Waterworks Free Chlorine Check Ultra High II (WW Ultra II) test strip (Rock Hill, SC); and, 5) Precision Laboratories active chlorine test strip (Precision High) (Cottonwood, AZ).

Serim was used by: immersing the indicator in the chlorine solution for 2 seconds, removing the test strip, shaking off excess solution, waiting 90 seconds, and then comparing the color at the center of the indicator pad to the color comparator chart. The Serim color ranged from blue to yellow, with a distinctive green and yellow color indicating 500 mg/L concentrations (indicator colors for 100, 200, 350, 500, and 750 mg/L). InstaTest was used by: immersing the strip in solution for 2 seconds, and holding the pad level to the color chart within 30 seconds. The color chart ranged from pink to purple (color indicators for 0, 50, 100, 250, 500, and 800 mg/L). WW Ultra I was used by dipping the test strip in solution for 1 second, waiting for 30 seconds, and then comparing to the comparator chart (color indicators for 0, 25, 100, 200, 300, 400, 500, 750 mg/L). WW Ultra II followed the same instructions as WW Ultra I, with a wait time of 60 seconds (color indicators for 0, 25, 50, 200, 500, 800, 1100, 1500, 2000 mg/L). Both the WW Ultra I and WW Ultra II had a light to dark blue color range. Precision High was used by dipping the pad in the solution for 1 second, shaking off excess solution, waiting for 60 seconds, and then comparing to the blue to brown color comparison range (color indicators for 0, 50, 100, 250, 500, and 1,000 mg/L).

### Usability Testing

In addition to the laboratory testing, ten volunteer participants used each method and provided feedback on usability. Volunteers were recruited through flyers posted on Tufts University campus and emailed out to the students of several academic departments. All participants were aged 18 years or older and gave written, informed, consent of their participation. In consent forms, and verbally at the beginning of participation, volunteers were informed that they could end their participation at any time without penalty. Participants followed written manufacturer instructions (with additional researcher-written instructions for cylinder and pipette dilutions) and used each test method to measure in duplicate 0.05% and 0.5% stabilized NaOCl solutions. Sample concentrations were unknown to participants. After using all the test methods, participants completed a questionnaire including information on prior laboratory experience, relative difficulty of test procedures, and confidence in results. Participants were also asked to indicate which method was easiest and which provided results they were most confident in. Lastly, participants were asked to identify which method they would recommend in different contexts and invited to provide open-ended comments on test method procedures. No identifying information was collected on forms filled out by volunteers during their participation. All volunteer group activities were approved by the Social, Behavioral, and Educational Research Institutional Review Board at Tufts University.

### Cost

Test method costs were calculated by adding fixed equipment and consumable reagent costs from manufacturer websites in June 2015, calculated for a total of 100 and 1,000 tests. The cost of Digital Titration, Pooltester, and Color Wheel methods include the price of equipment required to pipette and dilute solutions. Digital Titrations and pipette dilutions include the price of a pipette, pipette tips, and a 125 mL Erlenmeyer flask. Cylinder dilutions include the price of two graduated cylinders and a 500 mL beaker. The costs do not include shipping or handling, and were found, where applicable, on manufacturer’s United States websites. Therefore, costs may vary according to the geographic region in which they are purchased.

### Analysis

Data was entered into Microsoft Excel 2011 (Microsoft Corporation, Redmond, WA) and analysis performed in Excel and Stata 13.3 (StataCorp, College Station, TX). To assess accuracy, the average measurement error was calculated in reference to the Digital Titration method, and data was stratified into “low measurement error” of <10%, “medium measurement error” of 10–25%, and “high measurement error” of >25%. The Wilcoxon rank sum test, a nonparametric test for equality between samples, was used to assess whether there were significant differences between: 1) Digital Titration (as reference) and each other test method result from the same type of chlorine; and 2) stabilized NaOCl (as reference) and results from each other chlorine type, by test method. Precision for each method was assessed by calculating a standard error across quintuplicate readings. For volunteer testing, user measurement error was established by calculating the relative percent difference between the volunteer test results and the expected (laboratory tested) result for all participants’ readings.

The Digital Titration method was chosen as a reference method because: 1) titration methods are the standard for measuring chlorine concentration [22–24]; 2) the Digital Titration method is the most complete titration method we identified available for field use; and, 3) the method is not sensitive to pH variation (25). Stabilized NaOCl was chosen as the reference for chlorine type because the test methods were developed for use with this solution type [25,26].

Final criteria for recommendation of a test method included: 1) an accuracy <10% error across all five chlorine solutions; 2) standard error <0.01% as a measure of precision; and, 3) ease-of-use and cost appropriate to the context.

## Results

### Test Method Use

Overall, 16 individuals provided information about source chlorine compounds and/or test methods used during the 2014 West African EVD outbreak. It is not possible to calculate a response rate, as the initial email was widely forwarded to an unknown number of recipients. Based on these responses and online resources, we identified the 14 test methods described in methods for testing of Ebola-relevant chlorine concentrations (Table 1): two titration methods; four DPD methods; and, eight test strips.

### Accuracy and Precision

**Fig 1:**
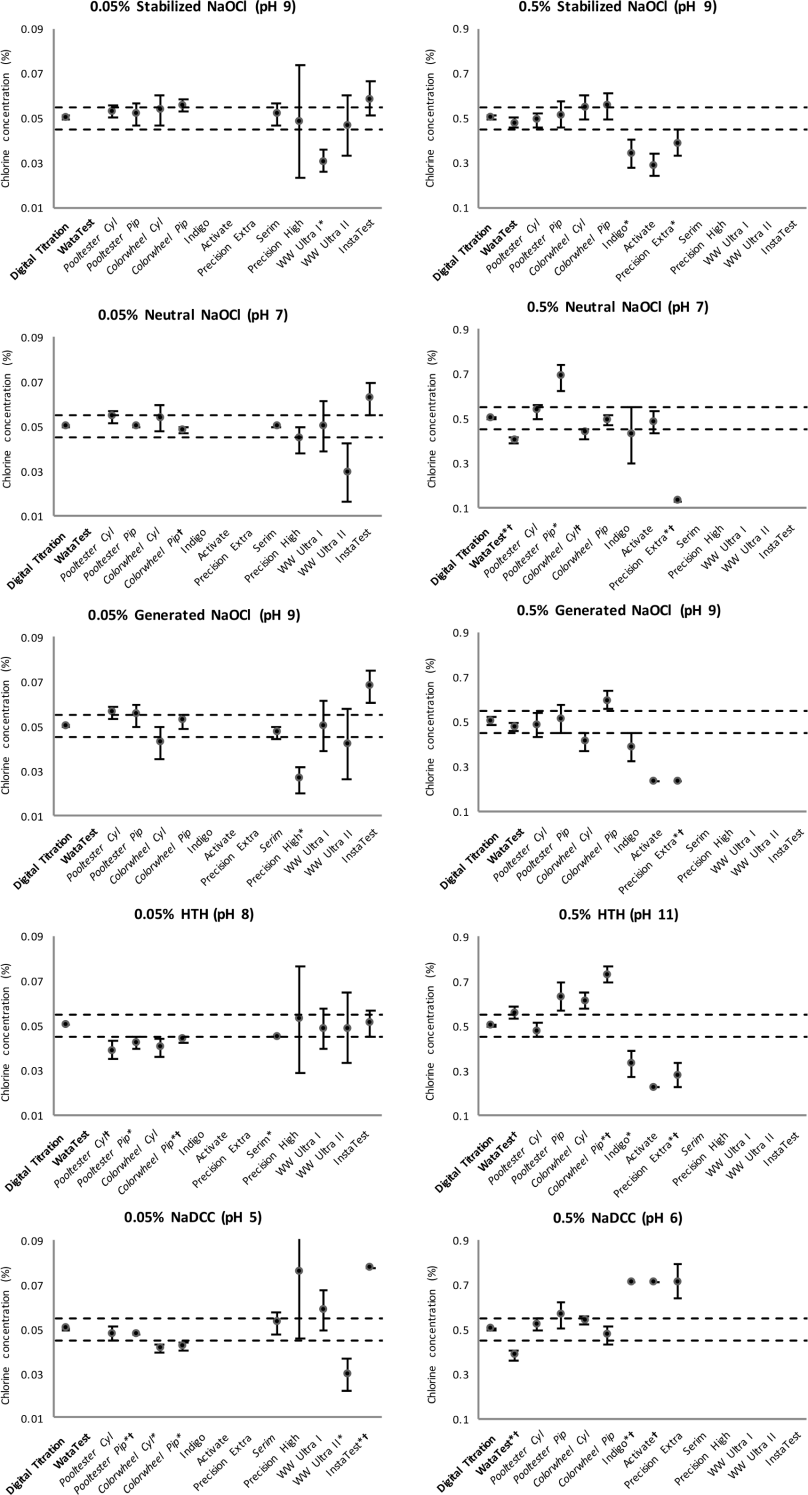
Average Measurement Error and Standard Error for 0.05% and 0.5% Chlorine Solutions. Dashed lines represent 10% acceptable margin of measurement error. Error bars represent standard error. *measurement error significantly different from digital titration (p<0.05); †measurement error significantly different from stabilized NaOCl (p<0.05). Bold: Titration Kits, Italics: DPD kits, Plain: Test Strips

**Fig 2:**
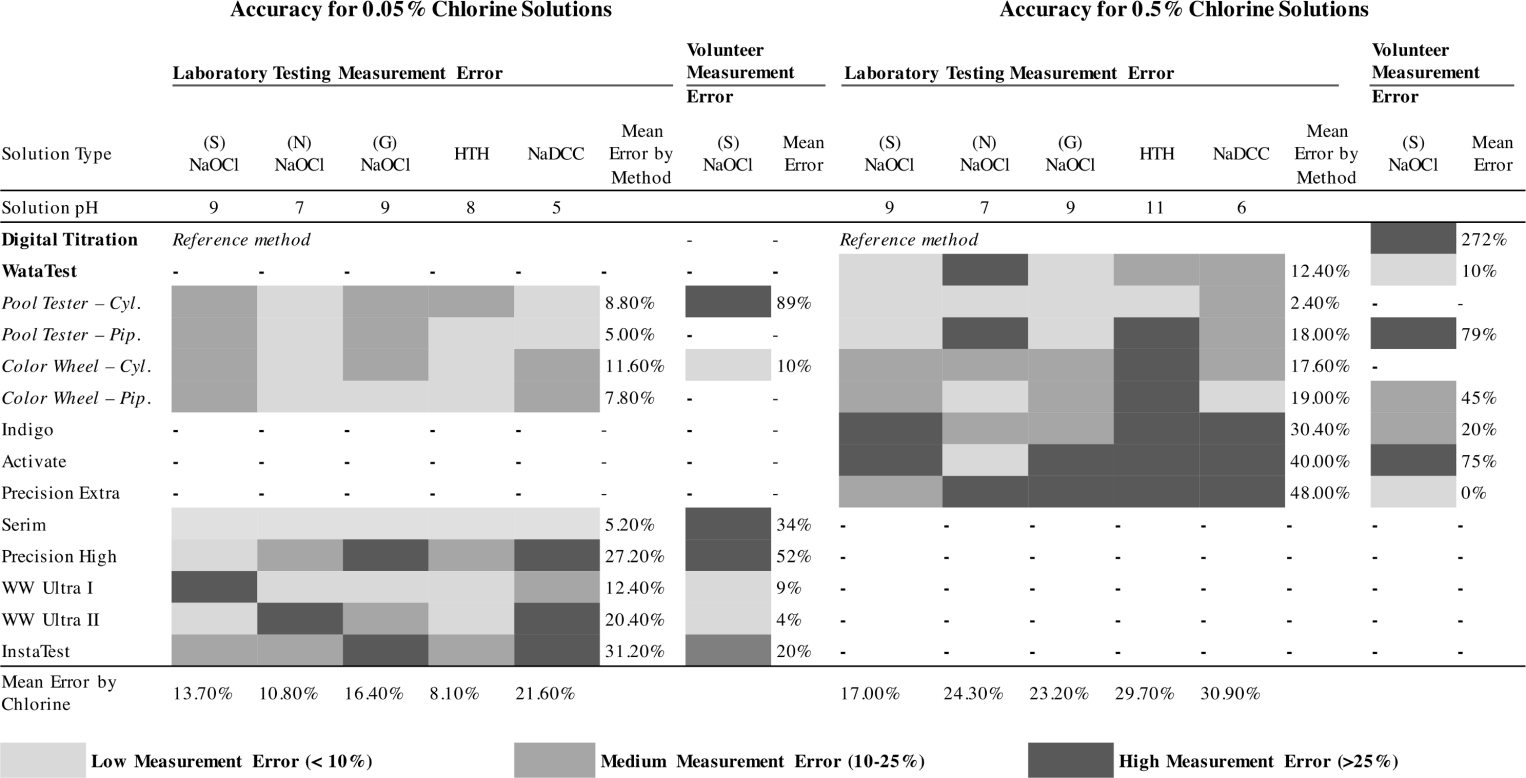
Accuracy in Laboratory and Volunteer Testing for 0.05% and 0.5% Chlorine Solutions Bold: Titration Kits, Italics: DPD kits, Plain: Test Strips

Chlorine solutions were manufactured and tested with the Hach digital titration kit to determine concentration. Solutions were then and adjusted until they were within 10% of the target value according to the Hach kit (0.05% or 0.5%). The Hach digital titration kit was used as a reference point for testing of other kits, as it was assumed to be the most accurate option available to field responders. Measurement error and standard error of test methods are presented in Fig 1 and Fig 2 to describe accuracy and precision of test kits, and results are presented below by concentration, chlorine source compound, and test method used.

#### 0.05% Chlorine Solutions

At 0.05% chlorine, nine test methods were compared against the reference Digital Titration method: four DPD dilution methods and five test strips (Table 1, Table 2). Across all chlorine types, the mean measurement error of the nine test methods ranged from 5.2–31.2% of the measured concentration; the overall average measurement error of all test methods was 14.4%. The mean measurement error was 8.3% for DPD dilution methods (range 5.0–11.6%) and 19.3% for test strips (range 5.2–31.2%). The DPD methods with pipette dilution were more accurate (average measurement error 6.4%) than the cylinder dilution methods (10.6%). Only one method, Serim test strip, was within 10% measurement error for all five chlorine solutions.

**Table 2:** Test Methods Cost.

Test	Equipment Cost	Consumable Cost	Cost per 100 tests	Cost per 1000 tests
Digital Titration	$426.24	$1.79	$608.92	$2,219.92
WataTest	$16.6	$0.17	$33.20	$186.20
Pooltester (Cylinder Dilution)	$35.12	$0.15	$50.12	$185.12
Pooltester (Pipette Dilution)	$266.24	$1.84	$282.2	$1,938.20
Color Wheel (Cylinder Dilution)	$83.48	$0.11	$94.17	$193.17
Color Wheel (Pipette Dilution)	$311.59	$0.38	$322.28	$664.28
Indigo	-	$0.20	$20.00	$200.00
Activate	-	$0.32	$32.24	$320.24
Precision Extra	-	$0.14	$14.00	$140.00
Serim	-	$0.19	$18.50	$189.50
Precision High	-	$0.14	$14.00	$140.00
WW Ultra I	-	$0.30	$29.98	$299.98
WW Ultra II	-	$0.30	$29.98	$299.98
InstaTest	-	$0.37	$37.00	$370.00

In neutralized NaOCl solution, no test method had significantly different results as compared to Digital Titration (p<0.05). In generated NaOCl solution, Precision High test strip results were significantly different (p = 0.030); and in stabilized NaOCl solution, WW Ultra I test strip results were significantly different (p = 0.005). In HTH solution, Serim test strip (p = 0.008), Pooltester with pipette dilution (p = 0.005), and Color Wheel with pipette dilution (p = 0.008) had significantly different results. In NaDCC solution, WW Ultra II test strip (p = 0.008), InstaTest test strip (p = 0.005), Color Wheel with cylinder dilution (p = 0.008) and pipette dilution (0.009), and Pooltester with pipette dilution (p = 0.007) had significantly different results.

One test strip, InstaTest, had significantly different results in NaDCC solution, as compared to stabilized NaOCl solution (p = 0.049). Color Wheel with pipette dilution had statistically different results in neutralized NaOCl, HTH, and NaDCC solutions (p = 0.016, 0.024, 0.011, respectively).

Pooltester with cylinder dilution was significantly different in HTH solution (p = 0.044).

Across all chlorine types, standard error ranged from 0.001–9.018% of the chlorine concentration (Fig 1). Digital Titration was the most precise test method (average standard error 0.001%), followed by DPD dilution methods overall (0.003%), and test strips overall (0.010%). The DPD methods with pipette dilution were more precise (0.002%) than the cylinder dilution methods (0.004%). Test strip standard error ranged from Serim (0.002%) to Precision High (9.018% chlorine).

#### 0.5% Chlorine Solutions

At 0.5% chlorine, eight test methods were compared against the reference Digital Titration method: one titration method, four DPD methods, and three test strips (Table 1, Table 2). Across all chlorine types, the mean measurement error for the eight test methods ranged from 2.4–48.0% and the overall average measurement error of all test methods was 20.0%. The mean measurement error was 12.4% for the WataTest titration method, 13.7% for DPD dilution methods (range 2.4–19.0%), and 39.0% for test strips (range 30.4–48.0%). The DPD methods with pipette dilution were less accurate (average measurement error 18.5%) than the cylinder dilution methods (10.0%). No test method was within 10% measurement error for all five chlorine solutions.

There were statistically significant differences between Digital Titration results and results from the following test methods and solutions: WataTest in neutralized NaOCl (p = 0.008) and NaDCC (p = 0.007); Pooltester with pipette dilution in neutralized NaOCl (p = 0.008); Color Wheel and pipette dilution in HTH (p = 0.008); Indigo in stabilized NaOCl (p = 0.026), HTH (p = 0.008), and NaDCC (p = 0.005); Activate in stabilized NaOCl (p = 0.013), generated NaOCl (p = 0.005), HTH (p = 0.005), and NaDCC (p = 0.005); and, Precision Extra in stabilized NaOCl (p = 0.044), neutralized NaOCl (p = 0.005), generated NaOCl (p = 0.005), and HTH (p = 0.007).

There were also statistically significant differences from results in stabilized NaOCl solutions and results from the following test methods and solutions: WataTest with neutralized NaOCl solution (p = 0.008), HTH (p = 0.033), and NaDCC (p = 0.037); Color Wheel with cylinder dilution with neutralized NaOCl (p = 0.044); Color Wheel with pipette dilution with HTH (p = 0.045); Indigo test strip with NaDCC (p = 0.005), Activate with NaDCC (p = 0.004), and Precision Extra with neutralized NaOCl (p = 0.005), generated NaOCl (p = 0.049), and HTH (p = 0.016).

Across all chlorine types, the precision ranged from standard error 0.008–0.062% of the chlorine concentration (Fig 2). Digital Titration was the most precise test method (average standard error 0.008%), followed by WataTest (0.019%), DPD dilution methods overall (0.040%), and test strips overall (0.420%). The DPD methods with pipette dilution were less precise (standard error 0.035%) than the cylinder dilution methods (0.049%). Test strip precision ranged from Activate as the most precise (standard error 0.020%) to Indigo (standard error 0.062%) as the least precise.

### Usability Testing

Usability testing was conducted to assess the accuracy and precision that volunteers were able to achieve with each kit and to gather their impressions about the kits. Ten Tufts University undergraduate and graduate students participated in the volunteer testing group. Of the participants, 70% (7/10) reported some general lab experience. When asked about specific experience with testing for water quality parameters, 40% (4/10) reporting beginner-level experience and 20% (2/10) intermediate-level.

For testing of 0.05% stabilized NaOCl solutions, mean user measurement error was smallest for the WW Ultra II test strips (4%), followed by WW Ultra I test strip (9%), and Color Wheel with cylinder dilution (10%) (Table 2). Measurement error was greatest for Pooltester with cylinder dilution (89%). User precision was greatest for the Precision High test strip (1.5% difference between two measurements), and lowest for the Pooltester with cylinder dilution (176% difference).

For testing of stabilized 0.5% NaOCl solution, user measurement error was smallest for the Precision Extra test strip (0% measurement error), followed by WataTest (10%), and the Indigo test strip (20%). User measurement error at this concentration was highest for Digital Titration (272%). User precision was greatest for the Indigo test strip (3.7% difference between two measurements), followed by the WataTest (8.67%), and the Pooltester with pipette dilution (12%). Precision was lowest in Digital Titration (182% difference).

When asked to describe the difficulty of testing on a scale from 1–5, participants rated Serim test strips as easiest (mean = 1.33) and Digital Titration as most difficult (mean = 5.00). Participants appreciated the clear instructions and ease of differentiating color results on the Serim test strip. Participants also noted that the instructions for Digital Titration were difficult to understand, there were too many different reagents, and that the many steps left room for error. Despite rating it the most difficult, participants stated they would prefer using Digital Titration as they felt once you learned it, it could be the most accurate.

### Cost

Including fixed and consumable materials, the total cost of performing 100 tests ranged from $33–609 for titration methods, from $50–322 for DPD dilution methods, and from $14–37 for test strip methods (Table 2). The total cost of performing 1,000 tests ranged from $95 for the Pooltester with cylinder dilution to $1,962 for Digital Titration.

## Discussion

Confirming chlorine solution concentrations is essential for quality control to ensure proper disinfection in EVD outbreaks. Test methods appropriate for these solutions have not previously been evaluated, and responders questioned which methods are accurate, precise, easy to use, and affordable. We tested 14 different test methods across five solutions at 0.05% chlorine and 0.5% chlorine concentrations. The Hach Digital Titration kit was used as a reference point for testing of other kits, as it represents the most accurate option available to field responders. It was also the most precise of the kits tested, followed in accuracy and precision by DPD dilution test methods. In general, with one exception, test strip methods had low accuracy and precision. Results for many test methods varied by chlorine solution. Overall, Digital Titration and Serim test strips produced consistently accurate and precise results across all chlorine solutions. Volunteers reported the Serim test strip to be easiest to use, while the Hach Digital Titration method was most difficult. Costs for the test methods varied from $19 for 100 Serim test strips to $609 for the equipment and consumables necessary for 100 tests using Digital Titration. Based on these results, we consider: 1) the role of chlorine solution pH in test method accuracy; 2) the appropriate balance between accuracy, precision, ease-of-use, and cost in test method selection; and, 3) appropriate recommendations for measuring Ebola-relevant chlorine solutions in ETU and community contexts.

Across the five source chlorine compound solutions, we noted variations in measurement error with chlorine solution pH. This effect was less visible among 0.05% concentrations, as compared to 0.5% concentrations, likely because more dilute solutions have a more consistent pH. Overall, we found the effect of pH varied with test method: the Digital Titration method was insensitive to pH changes at both 0.05% and 0.5%; WataTest read low at pH 6–7, accurately at pH 9, and high at pH 11; the DPD dilution methods were insensitive to pH changes at 0.05%, but sensitive at 0.5%; the 0.05% test strip methods read inconsistently, except for Serim, which was accurate across all pH values, and the 0.5% test strip methods (on average) read low at pH 9–11, closer to accurate at pH 7, and high at pH 6.

We attribute variations in accuracy with pH to a combination of: 1) test method chemistry / pH adjustment, and 2) the amount of dilution in the sample. In the Digital Titration method, a powder sachet is added to acidify the solution to pH<4 prior to titration. According to a Hach representative, DPD is a pH-sensitive chemical, and Color Wheel DPD powder pillows contain three different buffers to ensure consistent performance across pH [27]. Conversely, the WataTest titration does not include pH adjustment, and, according to Palintest (manufacturer of the Pooltester), the Pooltester tablets contain only DPD, not additional buffers [28]. While we contacted test strip manufacturers to better understand differences in test chemistry, most reported test method mechanisms were proprietary. To ensure the most accurate and consistent results, we recommend using test methods that include pH adjustment or are insensitive to pH.

Overall, test strip methods, which are easiest to use and least expensive, were the least accurate and precise, which was likely due to both the impact of pH and difficulty distinguishing color indicators. Different portions of Waterworks test strips often seemed to be different colors, and the Indigo and Precision extra, Precision high, and Instatest strips had color scales that were difficult to distinguish. The Serim test strip far outperformed other test strips, partly because the color scale had very distinct colors from yellow to green to blue to indicate 350, 500, and 750 mg/L, respectively. This unique scale made it simpler to read results than the continuous color scales on other test strips.

Based on criteria of having <10% mean measurement error for accuracy across all five chlorine solutions and standard error <0.01% chlorine, we recommend the Digital Titration method for 0.05% and 0.5% solutions, and Serim test strips for 0.05% solutions. These two recommended methods have quite different ease-of-use and cost implications: the Digital Titration method was identified as the hardest to use and most expensive, while the Serim test strip was the easiest to use and least expensive. Serim test strips are limited to increments at 350 mg/L, 500 mg/L, and 750 mg/L. While results were accurate for testing 500 mg/L (0.05%), accuracy was not verified at other concentrations. For example, the strip might incorrectly read a 600 mg/L solution (20% difference) as 500 mg/L; this was not evaluated in our study. Thus, we recommend the Digital Titration method for trained personnel with resources who need very accurate results, the Serim test strip for sufficiently accurate quick checks, or a combination of both tests (such as digital titration weekly and Serim daily) in situations such as ETU’s where daily testing is conducted.

Lastly, responders may already be familiar with the DPD free chlorine residual test kits because they are commonly used for water quality testing in emergency response settings (20). Results from the Pooltester and Color Wheel methods were potentially satisfactory, although they did not meet the above criteria described above, and were inconsistent across chlorine solutions and dilution types. These inconsistencies are attributed to difficulties in accurately performing dilutions even under ideal conditions, and lack of pH adjustment in the Pooltester. DPD kits had an intermediate cost, ranging from $50.12 for the Pooltester with cylinder dilution to $322.28 for the Color Wheel with pipette dilution. If responders are trained on these kits and there are chlorine-demand free water and supplies available for dilution, these methods might be used if alternatives are not available. However, given the variability observed even under ideal conditions we only cautiously recommend these tests for Ebola-relevant chlorine concentrations.

Additionally, we recommend further development of test strips available for 0.5% concentrations that have distinctive indicator colors and are robust to pH variation. In the interim, Serim test strips can be used for rapid assessment of 0.5% concentrations by diluting samples 10:1 with chlorine demand-free water.

Our research was limited by potential bias in laboratory measurements and application to Ebola-relevant contexts. Many results were dependent on visual color matching which may vary among users, and chlorine concentrations were unblinded to the researcher. Volunteers participating in usability testing were all well-educated university students, and many had prior laboratory and water quality testing experience, which may bias the results of tests to appear easier to use. Additionally, it is likely that conditions in the field are more variable than those we found in the lab, and this could result in greater variability and less accuracy for each test. For example, tests were performed on solutions produced with chlorine-demand-free waters, and do not account for variation in water that may alter total and free chlorine residual measurements. The use of impure water than exerts a chlorine demand will introduce variability into both chlorine production, and into the dilution process used for DPD tests. Lastly, chlorine solution efficacy on Ebola or Ebola surrogates was not assessed and the potential impact of variations in concentration on the Ebola virus is outside the scope of this work. Research on the inactivation threshold of the Ebola virus when exposed to chlorine solutions is in progress by several laboratory groups, however our research was intended to explored the fitness of tests in use for chlorine in the ranges currently used by responders and recommended by international agencies by applying a standard of 10% accuracy. While these recommendations are unlikely to change in the near term, these results should be re-confirmed if recommendations change.

Despite these limitations, we believe that these data represent a valuable estimation of the performance of these methods to test chlorine solutions at concentrations relevant for Ebola response. Our results downselect the available test methods that perform well in the laboratory setting at current Ebola-relevant concentrations. Further research is needed, in the field, to use these downselected test kits in field conditions to further refine recommendations. We have provided our results to responders, and are currently working with them to produce this field research.

The magnitude of the 2014 EVD outbreak in West Africa resulted in increased use of 0.05% and 0.5% chlorine concentrations in both ETU and community settings. In our work, we identified two potential methods for accurately and precisely testing chlorine solutions used in EVD response: a Digital Titration method that requires trained personnel and resources, and an easy-to-use, inexpensive test strip. We recommend future development of additional easy-to-use, inexpensive test strips for use in EVD response.

## Supporting Information

S1 Data Supporting information data (XLSX)
